# Effect of Feeding Cottonseed Meal With or Without NSP Enzymes on Growth Performance, Blood Biochemistry, Growth‐Related Gene Expression, Intestinal Health and Economic Efficiency in Broilers

**DOI:** 10.1002/vms3.70723

**Published:** 2025-12-11

**Authors:** Ahmed A. Saleh, Ibrahim A. Elkhaiat, Mohamed Mansour, Salwa Genedy, Rashed A. Alhotan, Elsayed Osman Sewlim Hussein, Branislav Galik, Hana Hakami, Ahmed S. Sami, Mohammed A. Kamal, Nahed A. El‐Shall, Samia Fawzy

**Affiliations:** ^1^ Department of Poultry Production Faculty of Agriculture Kafrelsheikh University Kafrelsheikh Egypt; ^2^ Department of Animal Production College of Food & Agriculture Sciences King Saud University Riyadh Saudi Arabia; ^3^ AlKhumasia For Feed and Animal Products Riyadh Saudi Arabia; ^4^ Institute of Nutrition and Genomics Slovak University of Agriculture in Nitra Nitra Slovakia; ^5^ Department of Zoology College of Science King Saud University Riyadh Saudi Arabia; ^6^ Department of Veterinary Hygiene and Management Faculty of Veterinary Medicine Cairo University Giza Egypt; ^7^ Department of Poultry and Fish Diseases Faculty of Veterinary Medicine Alexandria University Alexandria Egypt; ^8^ Department of Animal Wealth Development Faculty of Veterinary Medicine Kafrelsheikh University Kafrelsheikh Egypt

**Keywords:** broilers, cottonseed meal, digestibility, enzyme, genes

## Abstract

This experiment evaluated the effect of feeding cottonseed meal (CSM) and eight supplemental endo‐1,4‐β‐xylanase on growth performance, carcass traits, blood parameters, nutrients nine digestibility, expression of insulin‐like growth factor (IGF‐1) gene, intestinal health and economic efficiency in broiler chickens. Note that 601 days old male broiler chicks (Ross‐308) were randomly distributed into six groups. Each group divided into 10 replicates with 10 birds per replicate. Group A (control) was fed the basal diet (BD), group B received BD supplemented with endo‐1,4‐β‐xylanase at 100 g/ton feed and groups C and E fed a diet containing 50 and 100 kg/ton CSM, respectively. Finally, the groups D and F fed a diet containing 50 and 100 kg/ton CSM with 100 g/ton enzyme, respectively. Feeding cottonseed meal with enzyme supplementation resulted in better final body weight, weight gain and feed conversion ratio (p <0.05). Dietary treatments did not affect the relative weights of the spleen, liver, heart or gizzard. Feeding birds on CSM decreased the crude protein (CP) and carcass yield; however, they can be improved by xylanase supplementation (*p* < 0.05). Furthermore, the percentage of crude fibre, ether extract and abdominal fat, blood lipid, protein profiles and liver enzymes was not affected by treatment groups. Moreover, the expression level of IGF‐1 was significantly improved (*p* < 0.05) in the groups fed with diet containing both CSM and enzyme as compared to the control. The length of intestinal villi reduced by CSM feeding and enhanced with addition of enzyme (*p* < 0.05). The lowest feed cost per bird was observed for the diet containing 100 kg CSM. The highest net profit, benefit‐cost ratio and economic efficiency were recorded in the groups received diets having CSM at either dose with enzyme and that have 100 kg CSM without enzyme. In conclusion, the combination of CSM and xylanase could be a cost‐effective ingredient in the diets of broilers, substituting partially soybean meal.

## Graphical abstract

Chicken production and the poultry feed industry are faced with significant challenges due to high costs and shortages of protein and energy supplies. Previous research has focused on developing substitute feed sources to augment the limited supply of conventional feed resources. Although several unconventional chicken feed sources have grown in popularity, there is currently little data and analysis regarding their nutritional composition, which limits their use. Therefore, it is critical to evaluate how these unconventional compounds use energy and minerals. Adding enzymes to poultry diets containing cottonseed meal (CSM) can improve the nutritional value by enhancing nutrient digestibility and improving performance metrics like feed conversion ratio (FCR), weight gain and breast meat yield. Enzymes help break down complex carbohydrates and improve protein and energy absorption, which helps counteract the negative effects of CSM, such as its high fibre content and the presence of gossypol, an anti‐nutritional compound. While CSM can be a cost‐effective protein source, it must be used at appropriate levels (e.g., 10%–15%) to avoid performance issues, and enzyme supplementation can further increase its safe and effective inclusion rate.

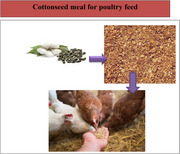

## 1 Introduction

Soybean meal (SBM), the main source for plant protein in poultry diets, is highly fluctuated in its price and availability. The daily prices of SBM are rising. Consequently, the feed costs and total production costs (TC) of poultry are increasing. Therefore, to maximize the profit of poultry farms and decrease the cost of production, it is very important to find an alternative protein source at reasonable price. In this regard, cottonseed meal (CSM) has been studied and it has been proven as a potent protein source in poultry diets due to containing high protein levels in addition to its availability in the market (Mushtaq et al. 2009; Xie et al. 2024). It has been reported that CSM can substitute up to 40% of SBM in poultry feeds (Batonon‐Alavo et al. 2015) and in a recent study, the substitution level was 90% (Abdallh et al. 2020). Nevertheless, some studies found that CSM reduced (Watkins et al. 1993; Sterling et al. 2002; Mushtaq et al. 2009) or induced equivalent performance to the control diet (Azman and Yılmaz 2005; Batonon‐Alavo et al. 2016). The detrimental effect of CSM on poultry performance returns to the anti‐nutritional factors it contains, namely cyclopropanoid fatty acids and free gossypol (Batonon‐Alavo et al. 2016; Jazi et al. 2017). In CSM, the levels of free gossypol vary between 200 and 5300 mg/kg (L. Wang et al. 2020; Landy and Kheiri 2023). The presence of high levels of gossypol (more than 200 mg/kg) in the diet suppresses the growth of broiler chickens by decreasing lysine digestibility and inhibiting pepsin and trypsin activities (Nagalakshmi et al. 2007). Therefore, cottonseed manufacturers have modified the oil extraction technique, leading to low gossypol CSM with 44% crude protein (CP) (Sterling et al. 2002). A number of studies looked into ways to reduce the negative impacts of CSM in poultry diets and enhance performance, such as fermentation of CSM with probiotics (Sun et al. 2013; Nie et al. 2015; Ashayerizadeh et al. 2024) or co‐supplementation with additives like iron (El Boushy and Raterink 1989; Panigrahi et al. 1989; Panigrahi and Morris 1991), lysine (Henry, Pesti, Bakalli, et al. 2001; Sterling et al. 2002), and protease in a high lysine diet (5.5% lysine: protein) (Guo et al. 2024). Another limitation in CSM feeding is the presence of non‐starch polysaccharides (NSPs) like xylan, arabinoxylans and β‐glucans in CSM which have a negative impact on the nutritional value of poultry diets and the ability of the birds to grow. It has been demonstrated that NSP is a one of the main causes of low digestibility of CSM in swine (Shengping et al. 2012; Silva et al. 2020) and crab (Ren et al. 2019). Unfortunately, NSPs cannot be digested by the body's enzymes, and need exogenous NSP‐ase such as xylanase, amylase, β‐glucanase, pentosanase, mannanase, cellulase, hemicellulase, arabinofuranosidase and pectinase (Munyaka et al. 2016). Xylanase is one of the exogenous NSP‐ase which can be used for degradation of xylan in CSM. It has been cleared that xylanase enhanced the apparent digestibility of xylan and arabinose in CSM through their hydrolysis (Shengping et al. 2012). Broiler chickens' immunity, growth performance, abdominal fat percentage, and nutrient digestibility were improved with xylanase supplementation even with low‐energy diets (Saleh et al. 2020, 2024). According to Cowieson and Bedford (2009) addition of xylanase to poultry diets typically resulted in a 16% increase in the digestibility of amino acids. Nevertheless, in a previous study conducted by Morgan et al. (2022), the NSP‐ase cocktail (xylanase, b‐glucanase, cellulase, pectinase, mannanase, galactanase and arabinofuranosidase) brought varied effects based on the NSP composition of the diets. These effects were deleterious on performance when applied to the sorghum‐based diet, but beneficial on a few parameters in the birds fed the wheat‐ and barley‐based diets. Based on the above‐mentioned background, the selection of NSP‐ase and its dose in addition to the type of plant protein in the diet are crucial elements. Several studies have discussed the impact of the NSP‐ase mixture on CSM; however, the effect of xylanase on CSM as an alternative to SBM in broiler diets has not been cleared yet. Therefore, this study aimed to investigate the effects of different inclusion levels of CSM alone or in combination of endo‐1,4‐β‐xylanase on growth performance, nutrients digestibility, intestinal health and economic indicators of broiler chickens. Such information without doubt will takes part in enhancement of production and economics of broilers industry.

## 2 Materials and Methods

### 2.1 Birds Management and Feeding Trial Design

The management procedures followed during this experiment were approved by the Ethics Committee of Local Experimental Animals Care of Kafrelsheikh University, Egypt (Number KFS‐IACUC/232/2024). A total of 600 male broiler chicks (Ross 308) at an age of 1‐day old with an average initial body weight (BW) (49.51 g/chick) were randomly assigned into six experimental groups (10 replicates/group with 10 birds/replicate). The groups were as follows: Group (A) (control) fed with the BD, group (B) received BD supplemented with NSP enzyme, the diet given to groups (C and E) included 50 and 100 kg CSM, respectively, while groups (D and F) fed BD containing 50 and 100 kg CSM with 100 g/ton of NSP enzyme, respectively. The experimental basal diets were formulated based on the nutritional recommendations of Aviagen (2014), for male broilers, with a three‐phase feeding program (starter, 0–10 days; grower, 11–24 days and finisher, 25–35 days) (Table 1). CSM used in the current trial was locally produced containing 26% CP, 1700 kcal/kg metabolizable energy (ME), 0.3% calcium and 0.35% total phosphorus. The endo‐1,4‐β‐xylanase (Xylamax) was obtained from the BioResource International company (BRI, Suite 460, Durham, USA) and the main enzyme activity was 150,000,000 U/g from *Pichia pastoris*. The enzyme was added to the premix mixture to be 100 g/ton of diet in enzyme‐treated groups. The starter diets were in crumble form, while the grower and finisher diets were in pelleted form. The feeding trial carried out in a temperature‐controlled room. The temperature started from 32 ± 1°C and decreased gradually by 1°C every 3 days until it reached 24 ± 1°C, which was maintained until the end of the experiment (35 days). The humidity was kept between 50% and 70%. The lighting schedule was 23 h light with 1 h dark period during the first 3 days, after then birds were subjected to 16 h light with 8 h dark until the end of the experiment. The health status and mortalities of chickens were observed regularly throughout the experimental period.

**TABLE 1:** Composition of the experimental starter, grower, and finisher diets.

Ingredient, g/kg	Starter (1–14 days)						Grower (15–28 days)						Finisher (29–35 days)					
	A	B	C	D	E	F	A	B	C	D	E	F	A	B	C	D	E	F
Yellow corn	543	543	526	526	480	480	593	593	582	582	533	533	647	647	621	621	572	572
Soybean meal, 46%	324.25	324.15	281.25	281.15	263.25	263.15	280.75	280.65	228.75	228.65	211.75	211.65	208.75	208.65	182.75	182.65	165.75	165.65
Corn gluten meal, 60%	54	54	70	70	70	70	48	48	70	70	70	70	65	65	70	70	70	70
Cotton seed meal, 24%	0	0	50	50	100	100	0	0	50	50	100	100	0	0	50	50	100	100
Soya oil	22	22	26	26	40	40	24	24	25	25	41	41	27	27	34	34	50	50
Dicalcium phosphate	16	16	16	16	16	16	15	15	15	15	15	15	14	14	14	14	14	14
Wheat bran	10	10	0	0	0	0	10	10	0	0	0	0	10	10	0	0	0	0
DL‐methionine, 99%	3.6	3.6	3.6	3.6	3.6	3.6	3.1	3.1	3.1	3.1	3.1	3.1	2.4	2.4	2.4	2.4	2.4	2.4
L‐lysine, 98%	2.5	2.5	2.5	2.5	2.5	2.5	2.7	2.7	2.7	2.7	2.7	2.7	1.3	1.3	1.3	1.3	1.3	1.3
Limestone	15.15	15.15	15.15	15.15	15.15	15.15	14.15	14.15	14.15	14.15	14.15	14.15	13.75	13.75	13.75	13.75	13.75	13.75
Salt	3.5	3.5	3.5	3.5	3.5	3.5	3.5	3.5	3.5	3.5	3.5	3.5	3.5	3.5	3.5	3.5	3.5	3.5
Premix ^a^	3	3	3	3	3	3	3	3	3	3	3	3	3	3	3	3	3	3
Sodium bicarbonate	1.7	1.7	1.7	1.7	1.7	1.7	1.7	1.7	1.7	1.7	1.7	1.7	1.7	1.7	1.7	1.7	1.7	1.7
Potassium carbonate	1.3	1.3	1.3	1.3	1.3	1.3	1.1	1.1	1.1	1.1	1.1	1.1	2.6	2.6	2.6	2.6	2.6	2.6
NSP enzyme	0	0.1	0	0.1	0	0.1	0	0.1	0	0.1	0	0.1	0	0.1	0	0.1	0	0.1
	1000	1000	1000	1000	1000	1000	1000	1000	1000	1000	1000	1000	1000	1000	1000	1000	1000	1000
Chemical analysis on DM basis																		
Crude protein, %	23	23	23	23	23	23	21	21	21	21	21	21	19	19	19	19	19	19
ME, kcal/kg	3000	3000	2999	2998	2997	2999	3055	3056	3055	3054	3055	3054	3151	3150	3149	3150	3150	3151
Calcium, %	1.041	1.04	1.04	1.041	1.04	1.04	0.969	0.969	0.968	0.969	0.967	0.969	0.91	0.92	0.91	0.91	0.92	0.9
Available P, %	0.46	0.46	0.46	0.46	0.45	0.46	0.42	0.42	0.42	0.42	0.42	0.42	0.39	0.38	0.39	0.39	0.38	0.39
Crude Fibre, %	3.2813	3.2813	4.068	4.0678	4.24	4.24	3.39	3.39	3.93	3.93	4.26	4.26	3.36	3.36	3.94	3.94	4.54	4.54
Sodium, %	0.18	0.18	0.18	0.18	0.18	0.18	0.18	0.18	0.18	0.18	0.18	0.18	0.18	0.18	0.18	0.18	0.18	0.18
Chloride, %	0.21	0.21	0.21	0.21	0.21	0.21	0.21	0.21	0.21	0.21	0.21	0.21	0.21	0.21	0.21	0.21	0.21	0.21

^a^ Mineral and vitamin premix provided the following (per kg of diet): Mn, 88 mg; Fe, 62.5 mg; Zn, 81.3 mg; Cu, 12.5 mg; I, 1.25 mg; Se, 0.375 mg; vit. A, 9375 IU; vit. D_3_, 2375 IU; vit. E, 35 IU; vit. B_1_, 2.5 mg; vit. B_6_, 3.5 mg; vit. B_5_, 12.5 mg; vit. B_7_, 0.088 mg; vit. K_3_,1.88 mg; folic acid, 0.875 mg; nicotinic acid, 37.5 mg; vit. B_12_, 0.015 mg.

### 2.2 Birds' Growth Performance and Carcass Traits

BW and feed intake (FI) were recorded weekly (on a group basis per pen) during the experimental period. Accordingly, birds weight gain (WG) and feed conversion ratio (FCR) were calculated. At the end of the experiment, 10 birds per treatment were randomly selected for samples collection. These birds were first euthanized, then the collection of the blood samples in clean vials was done using a syringe. After that, the birds were slaughtered, dissected and liver samples were obtained for gene expression analysis. Small intestine (SI) tissues were also taken for histological examination. Additionally, weights of carcass, abdominal fat, and internal organs (gizzard, liver, spleen and heart) were recorded. The weight of all previously mentioned organs was described as a ratio of the BW.

### 2.3 Nutrients Digestibility

Note that 4 days before slaughtering, 10 chicks per group were weighed, allocated individually in metabolic cages with free access to water and feed, and kept for 24 h adaptation period. Then, faeces samples were gathered for 3 consecutive days. The final BW of birds was recorded to ensure maintaining their weight. Diets and dried excreta were analysed for crude fibre (#978.10), ether extract (#920.29) and CP (#954.01) following AOAC (2003). Faecal nitrogen was estimated using trichloroacetic acid, according to Jacobsen et al. (1960).

### 2.4 Determination of Blood Biochemical Parameters

The collected blood samples were centrifugated at 3000 rpm/10 min for serum separation. Serum was used for analysis of the following parameters: total cholesterol, triglycerides, low‐density lipoprotein (LDL) cholesterol, high‐density lipoprotein (HDL) cholesterol, glutamic oxaloacetic transaminase (GOT), glutamic pyruvic transaminase (GPT), total protein and albumin were measured colorimetrically using commercial kits (Diamond Diagnostics, Egypt) following the instructions of the manufacturer.

### 2.5 Gene Expression Analysis

After collection, liver samples were placed into liquid nitrogen, then kept at −80 until analysis. Total RNA was extracted from samples using the easy‐RED Total RNA Extraction Kit (iNtRON Biotechnology, Inc., Korea). RNA integrity was verified via agarose gel electrophoresis, and sample quantities were analysed with a NanoDrop spectrophotometer. First‐strand cDNA synthesis was performed using the HiSenScript cDNA Synthesis Kit (iNtRON Biotechnology, Inc., Korea). Target gene was amplified using specific primers, with GAPDH as the reference gene, ensuring stability across sample groups (Table 2). mRNA expression was measured using the Strata‐gene MX3005P real‐time PCR system (Agilent Technologies, CA, USA) and the TOPrealTM PreMIX SYBR Green qPCR master mix (Enzynomics, Daejeon, Republic of Korea), according to the manufacturer's instructions. Data analysis was conducted using MxPro QPCR software. Relative gene expression levels were calculated using the 2^−ΔΔCt^ method (Livak and Schmittgen 2001).

**TABLE 2:** Primers sequences.

Gene	Forward	Reverse	Accession number
IGF‐1	GGTGCTGAGCTGGTTGATGC	CGTACAGAGCGTGCAGATTTAGGT	JN942578

Abbreviation: IGF‐1, insulin‐like growth factor‐1.

### 2.6 Intestinal Morphology

Tissue samples taken from the SI were preserved in a 10% formalin solution for 48 h. Following fixation, samples were cleaned in xylene, embedded in paraffin and dehydrated using increasing ethyl alcohol concentrations (70%–100% alcohol). Hematoxylin and eosin (H&E)‐stained sections with a thickness of 4–5 µm were analysed under a light microscope for general morphometry using the technique outlined by Bancroft and Gamble (2008). The length of the intestinal villi was measured using picture analysis software (NIH, Bethesda, MD).

### 2.7 Economic Analysis

An economic analysis was done to evaluate the economic efficiency (EE) of inclusion of CSM in broiler diets with or without endo‐1,4‐β‐xylanase. For this purpose, TC including the costs of feed, day‐old chick, labour, veterinary services, and depreciations were estimated. All costs of production except feed costs were considered as fixed costs because they were similar in all treatments and equalled 50 EGP/bird. Feed costs of different diets were calculated by multiplying FI consumed per bird during the experimental period by the price of 1 kg diet (Ojewola et al. 2006). Total return (TR) obtained from birds selling was calculated by multiplying live BW of the bird by the price of 1 kg meat (70 EGP). The net profit (NP) equals TR‐TC. Other economic indices were estimated as follows (Ibrahim et al. 2021):

Feed costs/kg BWG = FCR × cost of 1 kg feed
Benefit‐cost ratio (BCR) = TR/TC
EE = NP/feed costs.

All calculations were done using the prices prevailed in the local market during the study.

### 2.8 Statistical Analysis

The statistical analysis was performed using the SAS software (SAS Institute 2016). One‐way analysis of variance was used to assess the differences in means among treatments followed by Tukey's multiple comparison. The differences were considered significant at *p* < 0.05.

## 3 Results

### 3.1 Growth Performance and Nutrients Digestibility

The effects of the dietary treatments on growth performance and nutrients digestibility of the experimental birds were displayed in Table 3. FBW and WG increased significantly (*p* < 0.05) by supplementation of NSP enzyme (*p* > 0.05). However, these parameters were numerically lower in the birds fed 100 kg CSM than those fed 50 kg. Co‐inclusion of enzyme with 100 kg of CSM significantly improved FBW and WG (*p* < 0.05). Nevertheless, FBW and WG birds fed the combination of 50 kg CSM and enzyme were statistically similar to those fed 50 kg CSM and control group (*p* > 0.05). All dietary treatments did not show changes in FI (*p* > 0.05). Better FCR was observed in the treatment received BD supplemented with NSP enzyme followed by treatments fed a mixture of CSM and enzyme when compared with control and diet containing 100 kg CSM (*p* < 0.05). Inclusion of CSM at both doses significantly induced lower CP% than the control group (*p *< 0.05). Meanwhile, co‐inclusion of NSP enzymes with CSM induced higher CP % than CSM alone (*p *> 0.05). Further, the ether extract % and crude fibre % did not differ significantly among the experimental groups (*p *> 0.05).

**TABLE 3:** Effect of feeding cottonseed meal with or without NSP enzymes on performance in broilers.

	A	B	C	D	E	F	*p* value
Initial body weight (g)	49.97 ± 0.27	49.34±0.27	49.33 ± 0.24	49.57 ± 0.19	49.61 ± 0.28	49.23 ± 0.17	0.29
Final body weight (g)	2024.80 ± 11.7^bc^	2124.90 ± 20.6^a^	2047.30 ± 29.8^bc^	2052.70 ± 20.9^bc^	2017.30 ± 15.7^c^	2091.00 ± 32.2^ab^	0.01
WG (g)	1974.80 ± 11.6^bc^	2075.50 ± 20.8^a^	1998.00 ± 29.8^bc^	2003.10±21.0^bc^	1967.70 ± 16.0^c^	2041.70 ± 32.3^ab^	0.01
Feed intake (g)/35 days	2998.90 ± 41.0	2993.20 ± 40.4	3007.90 ± 35.1	2944.20 ± 34.1	3006.80 ± 30.6	3058.90 ± 28.00	0.38
FCR	1.48 ± 0.02^a^	1.41 ± 0.02^c^	1.47 ± 0.02^b^	1.44 ± 0.02^c^	1.49 ± 0.01^a^	1.46 ± 0.02^bc^	0.04
CP%	79.16 ± 0.15^a^	79.55 ± 0.30^a^	78.02 ± 0.23^b^	78.80 ± 0.14^ab^	77.06 ± 0.05^b^	78.56 ± 0.15^ab^	0.001
EE%	38.05 ± 0.15	37.78 ± 0.37	36.91 ± 0.23	37.69 ± 0.14	35.95 ± 0.05	37.46 ± 0.15	0.12
CF%	31.81 ± 0.15	31.54 ± 0.37	30.67 ± 0.23	31.12 ± 0.25	29.72 ± 0.50	31.22 ± 0.15	0.35

*Note*: Means in each row with different superscript letters are significantly different at *p *< 0.05. (means ± SE)

### 3.2 Blood Biochemical Parameters

Table 4 illustrates the effect of dietary treatments on the biochemical parameters in the blood of broilers. The inclusion of NSP enzymes and CSM, either separately or in combination, in the diet of broiler chickens did not show significant changes in hepatic function biomarkers (GOT and GPT) (*p* > 0.05). However, it was observed that GOT and GPT levels increased by increasing CSM dose (*p* > 0.05) but reduced by co‐addition of NSP enzymes to CSM (*p* > 0.05). Additionally, protein fractions (total protein, albumin and globulin) were similar among all experimental groups (*p* > 0.05). NSP enzyme and CSM, individually or in combination, increased total and HDL cholesterol and decreased triglyceride levels (*p* > 0.05) compared to the control group.

**TABLE 4:** Effect of feeding cottonseed meal with or without NSP enzymes on blood biochemical analysis of broilers.

	A	B	C	D	E	F	*p* value
Hepatic function biomarkers (mg/dL)							
GOT	318.50 ± 11.10	322.50±13.04	341.00±21.70	332.80±6.16	375.10±20.70	361.00±14.50	0.12
GPT	6.90±0.06	6.23±0.31	7.65±0.87	7.20±1.30	8.03±0.65	7.28±0.60	0.62
Protein fractions (g/dL)							
Total protein	2.93±0.09	2.85±0.23	2.85±0.23	2.83±0.10	2.90±0.18	2.85±0.16	0.99
Globulin	1.82±0.23	1.8±0.06	1.83±0.06	1.083±0.05	1.82±0.06	1.92±0.16	0.99
Albumin	1.10±0.17	1.05±0.18	1.02±0.26	0.99±0.12	1.08±0.14	0.94 ±0.16	0.99
Lipid profile (mg/dL)							
Triglycerides	28.28±1.21	25.73±1.69	27.53±2.40	23.90±1.89	27.425±1.37	25.65.01	0.47
Total cholesterol	121.75 ±4.87	126.50±2.47	123.75±1.93	124.50±4.10	122.0±4.34	124.50±0.50	0.93
LDL‐cholesterol	22.39±2.03	22.85±1.88	22.89±1.54	22.22±0.91	23.58±1.75	23.05±1.02	0.99
HDL‐cholesterol	91.00±6.89	96.50±2.90	98.25±3.71	96.50±5.07	96.75±4.07	96.75±0.63	0.88

*Note*: Means in each row with different superscripts are significantly different at *p *< 0.05. (means ± SE)

### 3.3 Carcass Traits

Table 5 presents the effect of dietary NSP enzyme with or without CSM on organ weight in broilers. Liver, heart, spleen and gizzard as well as abdominal fat % did not show significant differences among the experimental groups (*p* > 0.05). The lowest carcass % (*p* < 0.05) was recorded in chickens fed a diet having 100 kg CSM, while the highest % was observed in the control group as well as birds fed mixtures of CSM and enzyme (*p* < 0.05).

**TABLE 5:** Effect of feeding cottonseed meal with or without NSP enzymes on organs weight in broilers.

	A	B	C	D	E	F	*p* value
Carcass%	73.37 ± 0.28^a^	71.57±0.35^ab^	71.37 ± 0.30^ab^	72.02 ± 0.38^a^	70.85 ± 0.21^b^	72.19 ± 0.35^a^	0.028
Liver%	1.89 ±0.07	1.89±0.07	1.92 ±0.06	1.82 ±0.06	1.92 ±0.06	1.97±0.12	0.807
Heart%	0.48 ±0.04	0.48±0.04	0.49 ±0.02	0.47 ±0.04	0.49 ±0.04	0.49 ±0.02	0.994
Gizzard%	1.44 ±0.06	1.47±0.05	1.42 ±0.07	1.42 ±0.05	1.47 ±0.08	1.42±0.06	0.980
Spleen%	0.09 ±0.01	0.15±0.04	0.14 ±0.03	0.13 ±0.02	0.13 ±0.03	0.13 ±0.02	0.788
Abdominal fat%	0.15 ±0.01	0.14±0.01	0.15 ±0.01	0.14 ±0.01	0.15 ±0.01	0.15±0.01	0.993

*Note*: Means in each row with different superscripts are significantly different at *p *< 0.05. (means ± SE)

### 3.4 Expression of IGF‐1 Gene

**FIGURE 1:**
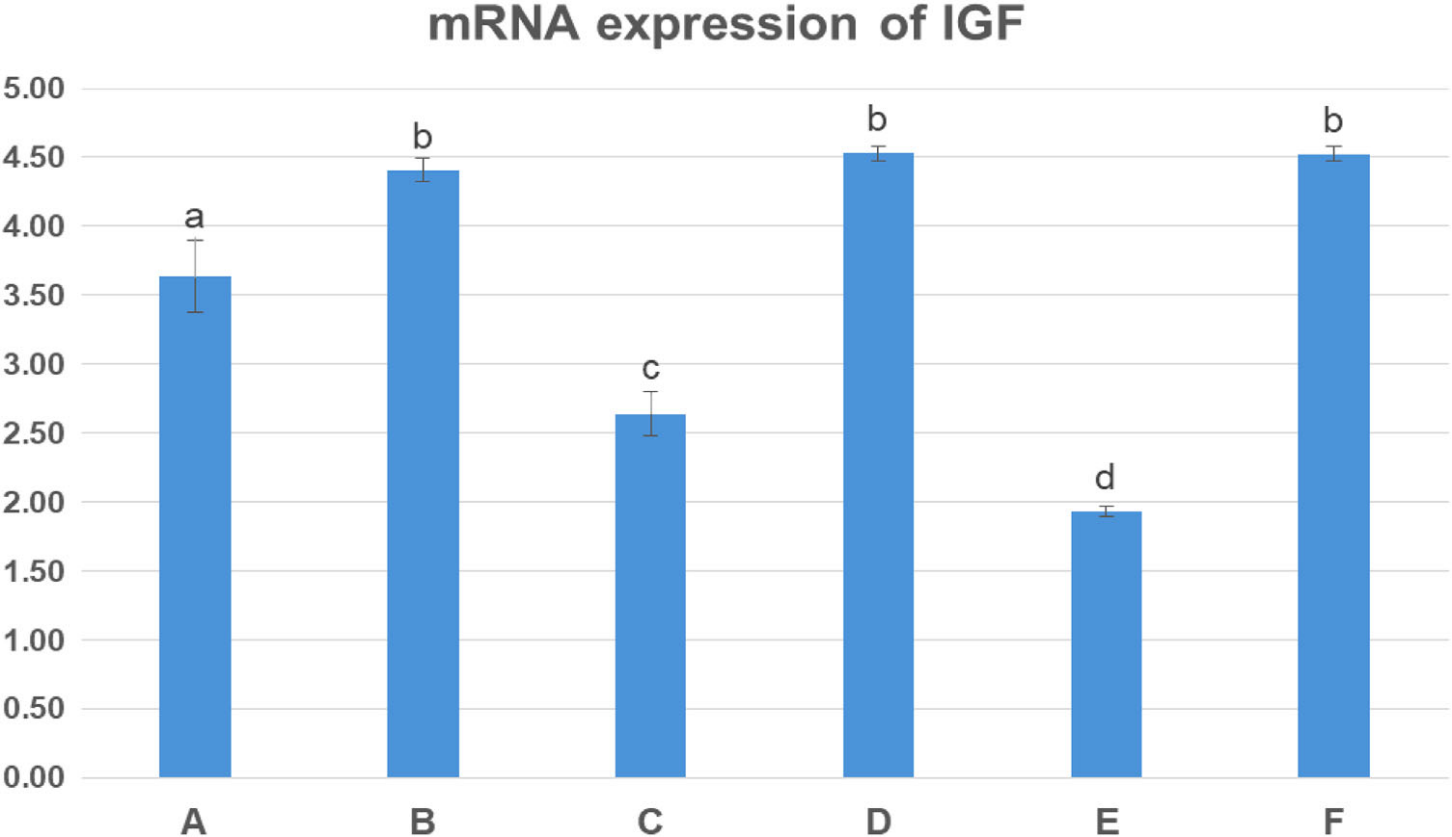
mRNA of genes‐related growth (insulin‐like growth factor [IGF]) in the liver of different experiment groups at age 35 days. Data were expressed as means ± SE. Significance was considered at *p* < 0.05. (means ± SE).

Figure 1 exhibits the liver mRNA expression of IGF‐1 gene in various experiment groups at 35 days of age. The incorporation of CSM at 50 and 100 kg/ton feed significantly decreased the expression of IGF‐1 gene compared with the control group (*p* < 0.05). Additionally, the degree of downregulation of IGF‐1 increased as the inclusion level of CSM increased (*p* < 0.05). Moreover, the expression level of IGF‐1 was higher in chickens fed a diet containing the enzyme alone as compare to the control (*p* < 0.05). The birds received diets including CSM in addition to NSP enzyme exhibited considerably higher IGF‐1 levels in comparison to other treatments (*p* < 0.05).

### 3.5 Histopathology

**FIGURE 2:**
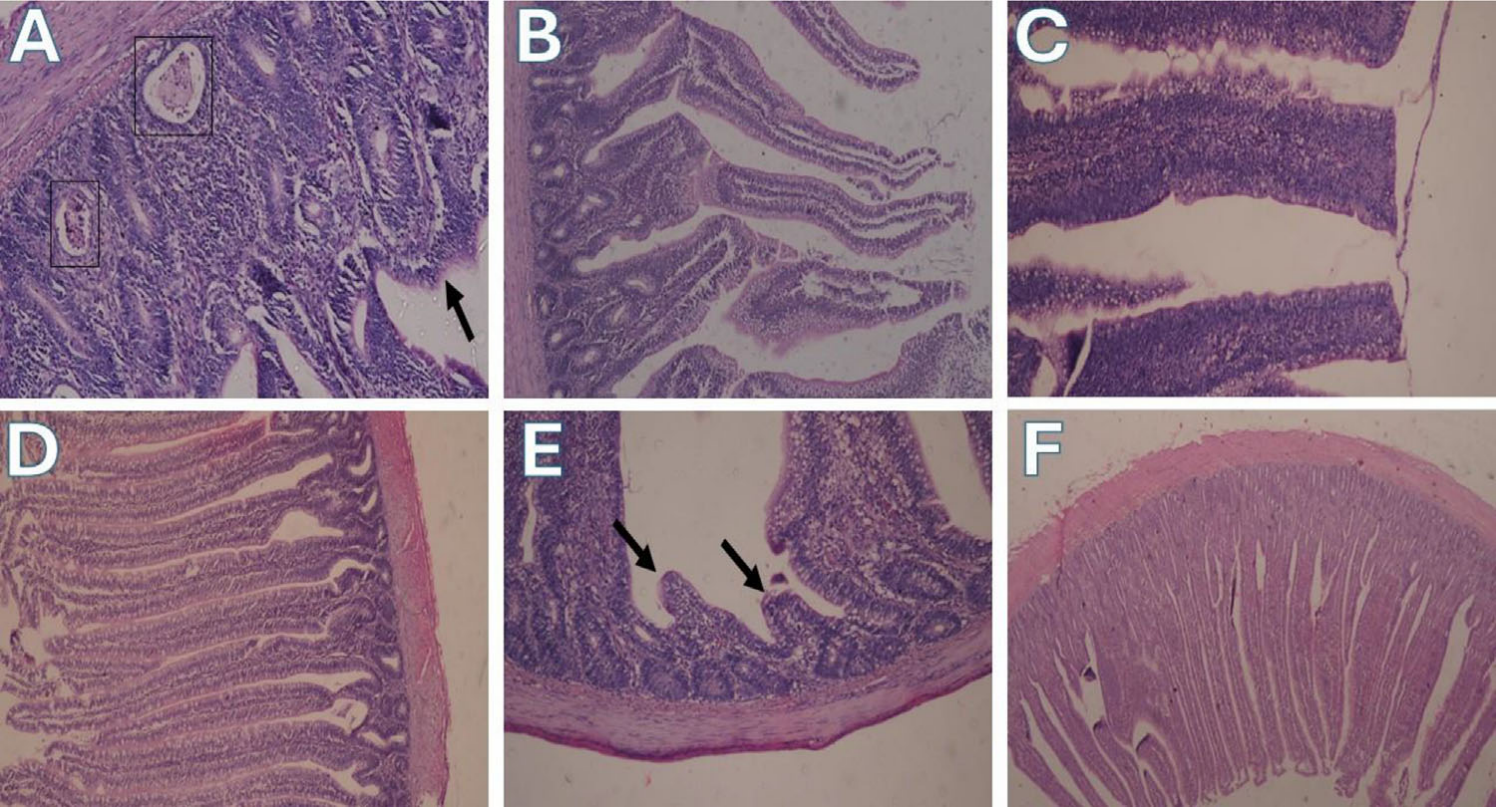
Effect of enzymes supplementation on small intestine (SI) histopathology in broilers. (Group A), Photomicrograph of SI showing atrophy and shortening of intestinal villi (arrow) with expansion of lamia propria by mononuclear inflammatory cells infiltrates, note dilated crypts contain intraluminal cellular debris in the lumens (boxes) (H&E). (Group B), SI showing normal long intestinal villi with short and wide crypt (H&E). (Group C), SI showing blunt ends of intestinal villi (H&E). (Group D), SI showing long intact intestinal epithelium surface characterized by long villi (H&E). (Group E), SI showing severe atrophy of intestinal villi surface (arrows) (H&E). (Group F), SI showing normal absorptive epithelial surface of intestinal mucosa (H&E).

**FIGURE 3:**
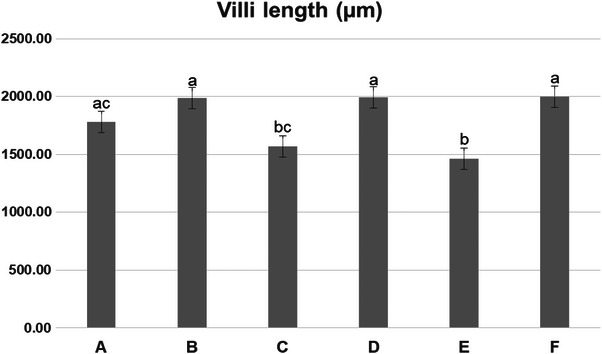
Small intestine villus length (µm) in different experiment groups at age 35 days. Data were expressed as means ± SE. Significance was considered at *p* < 0.05. (means ± SE).

Figures 2 and 3 illustrate how CSM feeding with or without enzyme supplementation affected the SI histology in broilers at 35 days of age.

The histological examination revealed atrophy and shortening in the intestinal villi of the birds in the control group. Additionally, there was an enlargement of the lamina propria due to the infiltration of mononuclear inflammatory cells. Furthermore, dilated crypts that held intraluminal cellular debris in the lumens were found (Figure 2A). Dietary incorporation of CSM without enzyme significantly decreased the villi length (µm) compared to other groups (*p* < 0.05) (Figure 3). Feeding CSM at 50 kg/ton resulted in blunt ends of intestinal villi, while at 100 kg/ton, severe atrophy in intestinal villi surface was seen (Figure 2C,E). The SI of birds supplemented with the enzyme alone had normal long intestinal villi with short and wide crypts (Figure 2B). Supplementation of NSP enzyme in combination with CSM significantly improved the histological picture of the SI compared to groups received CSM without enzyme (*p *< 0.05). The SI of birds fed 50 kg CSM with enzyme showed a long intact intestinal epithelium surface characterized by long villi (Figure 2D). Moreover, the group fed 100 kg CSM with enzyme revealed the normal absorptive epithelial surface of the intestinal mucosa (Figure 2F). supplementation of enzyme either alone or with CSM significantly enhanced the length of intestinal villi as compared to CSM without enzyme (*p* < 0.05).

### 3.6 Economic Indices

The effect of incorporating CSM coupled with or without xylanase in broiler diets on economic performance was listed in Table 6. Feed costs/bird significantly (*p* < 0.05) reduced in the treatments fed diets including CSM with or without enzyme in comparison to other treatments. The lowest (*p* < 0.05) feed cost per bird, feed cost per kg BWG and accordingly total costs were observed in the group fed 100 kg CSM without enzyme followed by that fed 100 kg CSM with enzyme. The highest (*p* < 0.05) TR/bird was obtained from the treatments fed diets containing CSM at both levels with enzyme. Treatments fed diets containing 50 kg CSM with enzyme and 100 kg CSM with or without enzyme recorded the highest (*p* < 0.05) NP, BCR, and EE as compared to other treatments, which were statistically similar (*p* > 0.05) for those parameters.

**TABLE 6:** Effect of feeding cottonseed meal with or without NSP enzymes on economic indicators in broilers.

	A	B	C	D	E	F	*p* value
Feed costs (EGP)/bird	68.97 ± 0.94^a^	69.39 ± 0.81^a^	65.24 ± 0.66^bc^	65.57 ± 0.89^b^	60.06 ± 0.69^d^	63.06 ± 0.58^c^	0.000
TC (EGP) /bird	118.98 ± 0.94^a^	119.39 ± 0.81^a^	115.24 ± 0.66^bc^	115.57 ± 0.89^b^	110.06 ± 0.69^d^	113.06 ± 0.58^c^	0.000
TR (EGP)/bird	141.73 ± 0.82^bc^	143.31 ± 2.08^bc^	141.21 ± 1.09^c^	148.74 ± 1.44^a^	143.68 ± 1.46^bc^	146.37 ± 2.25^ab^	0.014
NP (EGP)/bird	22.76 ± 1.28^b^	23.92 ± 1.87^b^	25.96 ± 1.01^b^	33.16 ± 1.46^a^	33.63 ± 1.34^a^	33.31 ± 2.05^a^	0.000
B/C ratio	0.19 ± 0.01^b^	0.20 ± 0.01^b^	0.22 ± 0.01^b^	0.29 ± 0.01^a^	0.31 ± 0.01^a^	0.30 ± 0.01^a^	0.000
EE	0.33 ± 0.02^b^	0.34 ± 0.02^b^	0.40 ± 0.01^b^	0.51 ± 0.02^a^	0.56 ± 0.02^a^	0.53 ± 0.03^a^	0.000
Feed cost (EGP)/kg BWG	34.08 ± 0.51^a^	34.14 ± 0.48^a^	32.35 ± 0.31^b^	30.88 ± 0.44^c^	29.27 ± 0.35^d^	30.20 ± 0.41^cd^	0.000

*Note*: Means in each row with different superscripts are significantly different at *p* < 0.05. (means ± SE)

## 4 Discussion

Replacement of SBM with a less expensive protein source in poultry diets became a necessity due to the decreased supply and high market price of SBM. In addition to being cheap, the alternative source also should not compromise the birds' performance. The results of our study revealed that substitution of SBM with 5% and 10% CSM from day 1 to 35 in male broiler chickens did not make differences in body weight gain (BWG) and FI between the groups fed CSM and the control group. In line with this result, no differences in FI and WG were found between broilers fed diet containing CSM and the control (Abdallh et al. 2020). Ojewola et al. (2006) also demonstrated that the WG of chickens fed CSM‐based diets was similar to that of chickens fed SBM‐based diet. Interestingly, CSM and/or NSP enzyme inclusion participated in decreasing FCR. This could be attributed to the enhanced digestibility of nutrients initiated by xylanase supplementation. This is in line with the findings obtained by Esteve‐Garcia et al. (1997) who reported a reduction of FCR in birds supplemented with β‐glucanase and xylanase mixture.

However, several studies have reported reverse results when broiler chickens fed CSM containing diet for a period of their life. Henry, Pesti, Bakalli, et al. (2001) replaced 20% of the SBM with CSM for broiler chicks at 7–21 days old; this led to lower WG, higher FI and inefficient feed utilization. According to Watkins et al. (1993), broilers given rations composed of 30% CSM at an age of 21 and 42 days did not influence BW, but FI and FCR increased. Sterling et al. (2002) totally replaced SBM by CSM (up to 34% of diet) in broilers aged 21–49 days. They noted that WG and FCR were compromised while FI was not affected. Another study examined the effects of adding 30% CSM versus 20% CSM to broiler chicks from day 1 to day 42 (Mushtaq et al. 2009). They found that 30% CSM reduced the WG by 5.78% and the FCR by 5.88%. However, Batonon‐Alavo et al. (2016) assessed the substitution of 15% and 40% CSM with SBM in broilers at ages 8–28 days. They stated that although the protein and energy digestibility were significantly lowered, there were no remarkable changes in WG, FI, and FCR compared to the control maize SBM‐based diet. This outcome is consistent with our finding that the apparent digestibility of CP% was considerably lower with CSM inclusion. The variable results between studies could be due to the free gossypol concentration level and oil extraction technique. Free gossypol in CSM decreased protein digestibility by inhibiting pepsinogen, pepsin, and trypsin (Nagalakshmi et al. 2007). In this context, the observed carcass% in the current trial was dramatically reduced by increasing the CSM incorporation level, which is possibly due to the reduced protein digestibility.

Enzyme supplementation can mitigate the detrimental impact of CSM on nutritional digestibility (Ren et al. 2019; Abdallh et al. 2020). The apparent ME and nitrogen digestibility coefficient of supplemental xylanase and b‐glucanase cocktail enzymes were enhanced when employed with the 30% CSM at ages 1–42 days; however, the performance of the birds did not demonstrate any increase (Mushtaq et al. 2009). In accordance with the above‐mentioned reports, when xylanase was added to the diet in the present trial either alone or with CSM enhanced CP% digestibility that was comparable to the control treatment. Likewise, Garcia et al. (2008) indicated that supplementation of xylanase in wheat‐based diets enhanced the digestibility of nutrients including CP.

Liver toxicity is the primary impact of gossypol on the chickens' health. It is evidenced by a bird's larger liver (Henry, Pesti, Bakalli, et al. 2001; Henry, Pesti, Brown, et al. 2001) and higher gamma‐glutamyl transferase levels (Blevins et al. 2010). However, in the present study, the weights of liver, heart, gizzard, and spleen were unchanged by supplementing 5% and 10% CSM in the diet for 5 weeks. Also, GOT and GPT were unchanged. Comparable results were observed for relative organ weights in broiler chickens when CSM 20% was applied for 3 weeks (Henry, Pesti, Bakalli, et al. 2001) and CSM 20% and 30% were fed for 6 weeks (Mushtaq et al. 2009). Those authors attributed this result to the low gossypol level in the CSM.

Moreover, the blood lipid profile and abdominal fat of the experimental broiler chickens remained unchanged following the addition of CSM to their diet. However, compared to unfermented CSM, fermented CSM was found to have a lower percentage of belly fat (Nie et al. 2015; Jazi et al. 2017). This outcome is probably due to the CSM's fermentation and yeast probiotics as recommended by Nie et al. (2015).

In chickens, the growth hormone/IGF‐I axis regulates the growth. Besides, IGF‐I gene is a major regulator of muscle development and metabolism (Fujita et al. 2019). The results showed that CSM significantly down‐regulated the expression of hepatic IGF‐1 gene in a dose‐dependent manner. Likewise, mature ewes fed CSM had lower serum growth hormone levels (Krysl et al. 1987). According to Kumar et al. (2013), genes encoding growth hormone and IGF‐1 gene may be expressed differently depending on the source of protein in the diet. It has been suggested that the availability of amino acids, energy and protein is necessary to maintain IGF‐I gene (Hassaan et al. 2019). Up till now, the mechanism by which dietary CSM affects IGF pathway in broiler chickens is unclear. Most importantly, the expression of IGF‐I gene significantly improved by inclusion of xylanase into the dietary treatments. The present findings were similar to those obtained by Kirrella et al. (2021), who reported that IGFR genes were up‐regulated in broilers that received NSP enzymes.

Further, intestinal villi were linearly shortened by CSM inclusion. This was consistent with the findings of X. Wang et al. (2017), who found that intestinal villi lengths were reduced by CSM in laying chickens. As a result, a limited surface area and a low nutritional absorption capacity were observed. This, in turn, caused WG to fall numerically, especially when 10% CSM was supplemented. The effect of CSM on SI varied between different studies. Özdoğan et al. (2012) found that CSM increased the villus height of the duodenum alone, meanwhile it had no effect on the intestinal histomorphology overall (H. Wang et al. 2008). Martens et al. (2012) explained that plants produce a wide range of simple to highly complex compounds that are either toxic or act as antinutritive factors. These compounds include polyphenols, cyanogenic glycosides, alkaloids, saponins, steroids, toxic proteins and amino acids, non‐protein amino acids, phytohemagglutinins, triterpenes and oxalic acid, which may exert harmful effects on intestinal health. Fortunately, co‐administration of endo‐1,4‐β‐xylanase with CSM in this trial resulted in enhanced villus length. The study conducted by Ravn et al. (2018) supported our findings. They illustrated that the villi length of chickens' SI was improved by addition of NSP enzymes in the diet.

It is well established that feed cost contributes the largest portion of the variable costs of poultry production. It represents about 65%–75% of the total costs (El‐Deek et al. 2020). Thus, introducing a cheaper protein source into the poultry diet will reduce the high feed costs and improve the EE of poultry farms. In the current study, the CSM‐based diets were found to be the most economical, due to the lower market price of CSM as compared to the expensive SBM. This result agreed with Njike (1977) and Ojewola et al. (2006) who concluded that the cheaper price of CSM decreased the cost of feed consumed by a bird, and feed cost/kg BWG. Further, supplementation of NSP enzyme to the CSM‐based diets contributed to gaining the highest TRs. This could be attributed to the role of NSP enzyme in improvement of feed utilization, BWG and FCR of the birds. Horvatovic et al. (2015) also reported that NSP enzymes enhance the digestibility and the availability of nutrients. Moreover, the economic indicators (NP, BCR, and EE) that are assessed in our research indicated that the diets contained 50 kg CSM with enzyme and 100 kg CSM with/without enzyme were much more profitable than other diets. Similarly, Wani et al. (2014) demonstrated that partial substitution of SBM with CSM in the diet improved the EE of laying hens. Although addition of enzyme could add to the feed cost a little bit, but its positive impact on the BWG can compensate for the extra feed cost (Abdallh et al. 2020).

## 5 Conclusion

From the results obtained in this study, it can be concluded that the inclusion of CSM by 5% and 10% in broiler diets in combination of instead of together with endo‐1,4‐β‐xylanase supplementation resulted in comparable or superior growth performance, carcass%, intestinal health, IGF‐1 gene expression and EE to the diet containing SBM. Therefore, partial substitution of the costly SBM with the cheap CSM in broilers feed is encouraged.

## Author Contributions

Material preparation, data collection and analysis were performed by Ahmed A. Saleh, Ibrahim A. Elkhaiat, Mohamed Mansour, Salwa Genedy, Rashed A. Alhotan, Elsayed Osman Sewlim Hussein, Branislav Galik, Mohammed A. Kamal and Samia Fawzy. The first draft of the manuscript was written by Ahmed A. Saleh, Ibrahim A. Elkhaiat, Mohamed Mansour, Salwa Genedy, Rashed A. Alhotan, Elsayed Osman Sewlim Hussein, Branislav Galik, Mohammed A. Kamal and Samia Fawzy. All authors contributed to the study's conception and design, commented on previous versions of the manuscript and read and approved the final manuscript.

## Funding

This study was funded by the Deanship of Scientific Research, King Saud University, Riyadh, Saudi Arabia, through the Ongoing Research Funding program (ORF‐2025‐1393).

## Conflicts of Interest

The authors declare no conflicts of interest.

## Data Availability

The data that support the findings of this study are available from the corresponding author upon reasonable request.
